# Structural Insights into the Specificity of 8-Oxo-7,8-dihydro-2′-deoxyguanosine Bypass by Family X DNA Polymerases

**DOI:** 10.3390/genes13010015

**Published:** 2021-12-22

**Authors:** Andrea M. Kaminski, Thomas A. Kunkel, Lars C. Pedersen, Katarzyna Bebenek

**Affiliations:** Genome Integrity and Structural Biology Laboratory, National Institute of Environmental Health Sciences, National Institutes of Health, 111 TW Alexander Dr., Bldg. 101, Durham, NC 27709, USA; andrea.kaminski@nih.gov (A.M.K.); kunkel@niehs.nih.gov (T.A.K.); bebenek@niehs.nih.gov (K.B.)

**Keywords:** Family X polymerases, 8-oxo-guanine (8OG), oxidized base damage, DNA repair, base excision repair (BER), nonhomologous end-joining (NHEJ)

## Abstract

8-oxo-guanine (8OG) is a common base lesion, generated by reactive oxygen species, which has been associated with human diseases such as cancer, aging-related neurodegenerative disorders and atherosclerosis. 8OG is highly mutagenic, due to its dual-coding potential it can pair both with adenine or cytidine. Therefore, it creates a challenge for DNA polymerases striving to correctly replicate and/or repair genomic or mitochondrial DNA. Numerous structural studies provide insights into the mechanistic basis of the specificity of 8OG bypass by DNA polymerases from different families. Here, we focus on how repair polymerases from Family X (Pols β, λ and µ) engage DNA substrates containing the oxidized guanine. We review structures of binary and ternary complexes for the three polymerases, which represent distinct steps in their catalytic cycles—the binding of the DNA substrate and the incoming nucleotide, followed by its insertion and extension. At each of these steps, the polymerase may favor or exclude the correct C or incorrect A, affecting the final outcome, which varies depending on the enzyme.

## 1. Introduction

Reactive oxygen species (ROS) generated as a product of aerobic cellular metabolism or by environmental factors such as pollution, ionizing and ultraviolet radiation may damage genomic DNA. Over time, the continuous onslaught of ROS-induced damage erodes genomic integrity, causing oxidative stress, which leads to disease and aging [1,2]. One of the most frequent DNA lesions caused by oxidative stress is 8-oxo-7,8-dihydro-2′-deoxyguanosine (8OG), resulting from hydroxyl radical addition to the C8 position of guanine [3,4]. The major tautomer of 8OG, at physiological pH, has a carbonyl group at the C8 position and is protonated at N7. Consequently, 8OG has dual coding potential. In the *anti* conformation, 8OG forms a correct Watson-Crick base pair with cytidine (*anti*) while rotation around the N-glycosidic bond to the *syn* conformation allows it to base pair with adenine (*anti*), through interactions with its modified Hoogsteen edge [5,6,7]. Given its elevated potential to pair with adenine, 8OG is highly mutagenic and, if not repaired, leads to G to T transversions, which are common somatic mutations associated with human cancer [8,9]. In addition to its well documented role in cancer, 8OG has been associated with the development of atherosclerosis and cardiovascular disease [1]. It has also been implicated in aging-related neurodegenerative diseases such as Parkinson’s, Huntington’s and Alzheimer’s (AD) [10,11,12]. Studies show that 8OG accumulates in brains of AD patients, and a notable decrease of MTH1 (MutT Homolog 1) and OGG1 (8-oxoguanine DNA glycosylase 1), two enzymes responsible for keeping the levels of 8OG low in human cells, has been observed in the brains of patients with sporadic AD [13,14]. The MTH1 hydrolase breaks down 8-oxo-dGTP to 8-oxo-dGMP, sanitizing the nucleotide pools and preventing 8OG incorporation into DNA [15,16], while oxidized guanines in DNA are primarily removed by base excision repair (BER) [17,18]. OGG1 glycosylase excises the 8OG base from the DNA duplex, but only when it is paired with cytidine [19,20]. Another glycosylase, MUTYH, recognizes the 8OG:dA mispair and excises the adenine [21]. In this case, the remaining 8OG will have another chance for repair after the single nucleotide gap initiated by the removal of A is filled by a DNA polymerase.

Thermodynamic measurements and structural NMR studies indicate that the 8OG:dA pair is more stable than the pair with dC [5,22]. In addition, the *syn* conformation of a template 8OG may be preferred, due to the potential clash between the bulky O8 carbonyl in the *anti* conformation and the sugar-phosphate backbone of the template DNA. Nevertheless, whether dAMP or dCMP is preferentially incorporated opposite a template 8OG is modulated by and depends predominantly on the DNA polymerase [23]. Structural studies reveal differences in the way DNA polymerases bind the lesion-containing DNA. Differential stabilization of the 8OG correct pair with dC or the mispair with dA in the nascent base-pair binding pocket (0 position) or at the template–primer terminus (−1 position) provides insights into polymerase preference for correct or incorrect insertion [24,25,26,27,28,29].

**Figure 1:**
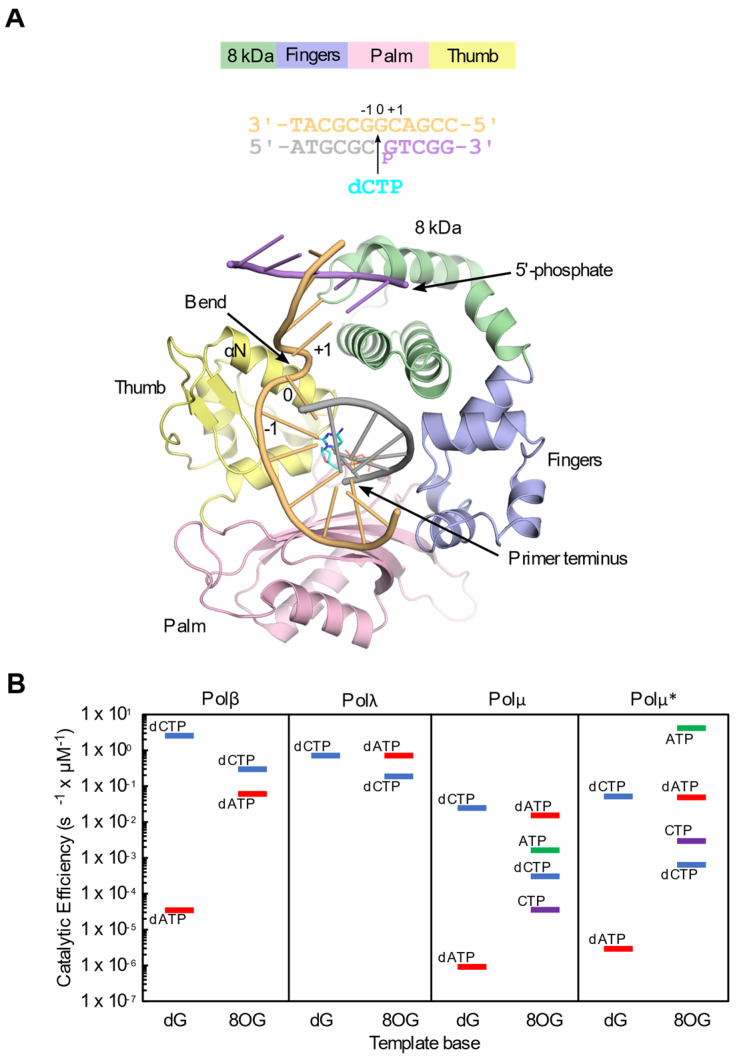
Comparison of catalytic efficiencies for nucleotide insertion by the Family X polymerases opposite undamaged template dG versus 8OG. (**A**). Diagram of domain architecture (top), gapped DNA substrates (middle) and global structure (bottom) of the Family X polymerase catalytic domain. Crystal structure of Polβ ternary complex with unmodified 1nt-gapped DNA substrate (PDB ID code 4KLD [32] and incoming nucleotide (cyan). The individual subdomains are colored as specified at top. Oligonucleotides are colored as follows—template in khaki, upstream primer in gray, and downstream primer in lavender. Locations of the 5′-phosphate, primer terminus (opposite the −1 position), template bend, and nascent base pair binding site (0 position) are indicated. (**B**). Kinetic parameters for deoxyribonucleotide (dCTP in blue, dATP in red) or ribonucleotide (CTP in purple, ATP in green) incorporation by Pols β [33], λ [34], and μ [35] in the context of a 1nt-gapped DNA substrate. Catalytic efficiencies for incorporation by Polμ were also normalized for experimentally determined cellular concentrations of nucleotide triphosphates [36] (calculated catalytic efficiency multiplied by nucleotide concentration, right panel, indicated by asterisk).

Here, we discuss how human repair polymerases, members of Family X (Pols β, λ and μ) bypass 8OG. A fourth member of Family X, terminal deoxynucleotidyl transferase (TdT), is present in human cells, but in contrast to its siblings, its localization and function is limited to lymphoid tissues [30]. Family X polymerases are small, monomeric enzymes devoid of 3′ to 5′ exonuclease activity. Their primary function is to fill small gaps in DNA, arising as intermediates during repair transactions. A characteristic structural feature of Family X polymerases is their DNA-binding 8 kDa subdomain, which facilitates gap-filling by allowing the polymerase to bridge both ends of the gap, where the 3′ and 5′ ends of the gap are bound by the polymerase and 8 kDa subdomains, respectively (Figure 1A) [31]. 

Polymerases (Pols) β and λ participate in BER. Polβ is the main polymerase in the short-patch BER pathway—which removes and replaces a single nucleotide—with Polλ playing a backup role in this process [37,38]. Long-patch BER involves the repair of 2–10 nucleotide stretches in DNA [39]. In BER initiated by MUTYH glycosylase, either Polβ or Polλ could insert dC or dA into the single-nucleotide gap, as neither has a marked preference during insertion opposite 8OG (Figure 1B). Insertion of dA would result in a futile repair cycle. However, it has been reported that the MUTYH-initiated repair preferentially proceeds through the long-patch BER pathway [40,41,42,43], and that Polλ, which discriminates against the 8OG:A mispair at the extension step [34], efficiently extends the dC:8OG pair [44], performing error-free bypass. Polλ, like Polµ, participates in the repair of double-strand breaks (DSBs) by non-homologous end joining (NHEJ) [45]. Along with TdT, they both are involved in the repair of programmed DSBs through a specialized form of NHEJ, known as V(D)J recombination, which is responsible for the maturation of immunoglobulins and T-cell receptors [46]. In addition, the ubiquitously expressed Pols λ and µ participate in general NHEJ, the primary pathway for repair of DSBs outside the S and G2 phases of the cell cycle [47].

The DNA ends of DSBs generated by ionizing radiation or UV light are seldom “clean”. Instead, damage clusters, including oxidized bases, persist close to break sites [48] due to the less efficient repair at the vicinity of the break [49]. Therefore, when repairing such complex DSBs, the NHEJ Pols µ and λ, may encounter 8OG in the template strand. In contrast to Pols λ and β, Polµ displays a strong preference for insertion of adenine, rather than cytidine opposite 8OG, though its preference for incorporation of dC opposite a normal dG is ~3 × 10^4^ -fold higher than for incorporation of A (Figure 1B) [35]. Due to Polµ’s propensity for ribonucleotide incorporation, it shows a similar ratio of preference for ATP insertion versus CTP (45-fold) opposite 8OG as for dATP versus dCTP (~50-fold). Considering that out of the eight nucleotides, the cellular concentration of ATP is the highest throughout the cell cycle [36], and that DNA ligase IV, which completes NHEJ by sealing the nick, prefers an rNMP rather than a dNMP on the 3′-terminus of the nicked DNA [50,51], the mutagenic incorporation of an AMP may be advantageous for the cell, providing for rapid and efficient repair of a cytotoxic, deleterious lesion like a DSB. In this case, the AMP:8OG mispair would have to be dealt with after the resolution of the DSB. Here, we review recent structural studies to discuss the molecular basis of the specificity for nucleotide incorporation opposite template 8OG by human Family X polymerases.

## 2. Fidelity of Template 8OG Bypass Differs among the Family X Polymerases

Each of the template-dependent Family X polymerases displays a unique pattern of template 8OG bypass fidelity. Among them, Polβ displays the highest fidelity, with a subtle (up to 5-fold) preference for correct insertion of dCTP rather than the mutagenic insertion of dATP opposite the 8OG in a single-nucleotide gapped single-strand break substrate (Figure 1) [33].The catalytic efficiency for incorporation opposite the template 8OG is only slightly decreased as compared to an undamaged template guanine. In contrast, Polλ displays a preference (up to 4-fold) for dATP insertion over dCTP [34]. dATP incorporation opposite the oxidized guanine lesion is performed with the same efficiency as that of dCTP opposite the undamaged guanine. Polμ, however, exhibits a strong preference (50-fold) for mutagenic incorporation of dATP opposite the 8OG over correct insertion of dCTP [35]. Insertion of dATP opposite the template 8OG is performed almost as efficiently as that of correct dCTP opposite undamaged guanine. Polμ, which only weakly discriminates against ribonucleotides [52], also displays a similar ratio of ATP versus CTP insertion (45-fold) opposite the 8OG. Given the high cellular concentrations of ribonucleotides [36]—that of ATP, in particular—Polμ’s propensity for insertion of ATP opposite 8OG [35] could provide an efficient means for this polymerase to resolve DNA double-strand breaks containing clusters of oxidized damage near the ends, though in a mutagenic manner (Figure 1). Ribonucleotides inserted during NHEJ can be efficiently ligated by DNA Ligase IV, even in the context of the template 8OG [35,51]. The Ribonucleotide Excision Repair pathway can effectively remove these ribonucleotides [53] once the DSB has been resolved, though it is unclear whether this occurs before or after OGG-1 or MUTYH-mediated repair.

## 3. Bypass of a Template 8OG Begins with DNA Substrate Binding

The structural means by which bypass of a template 8OG is carried out by the Family X polymerases has been thoroughly investigated. For all three polymerases, the distinct steps relevant to 8OG bypass have been characterized structurally (Table 1, Table 2 and Table 3). Following the initial binding of the lesion-containing DNA and the incoming nucleotide, bypass involves the insertion of the incoming nucleotide opposite the lesion (initial bypass) and a further extension step that seals the lesion in. For enzymes like Pols β and λ which participate in long-patch BER, this final step is an extension from the primer terminus opposite the template lesion. However, in the case of short-patch BER, or for polymerases like Polµ, whose role in NHEJ is limited primarily to inserting a single nucleotide, ligation would finalize bypass. Understanding the specificity of lesion bypass by these polymerases is to appreciate the differences in the interactions of the enzyme with its substrates at each of the steps leading to bypass and their importance to the final outcome.

**Figure 2:**
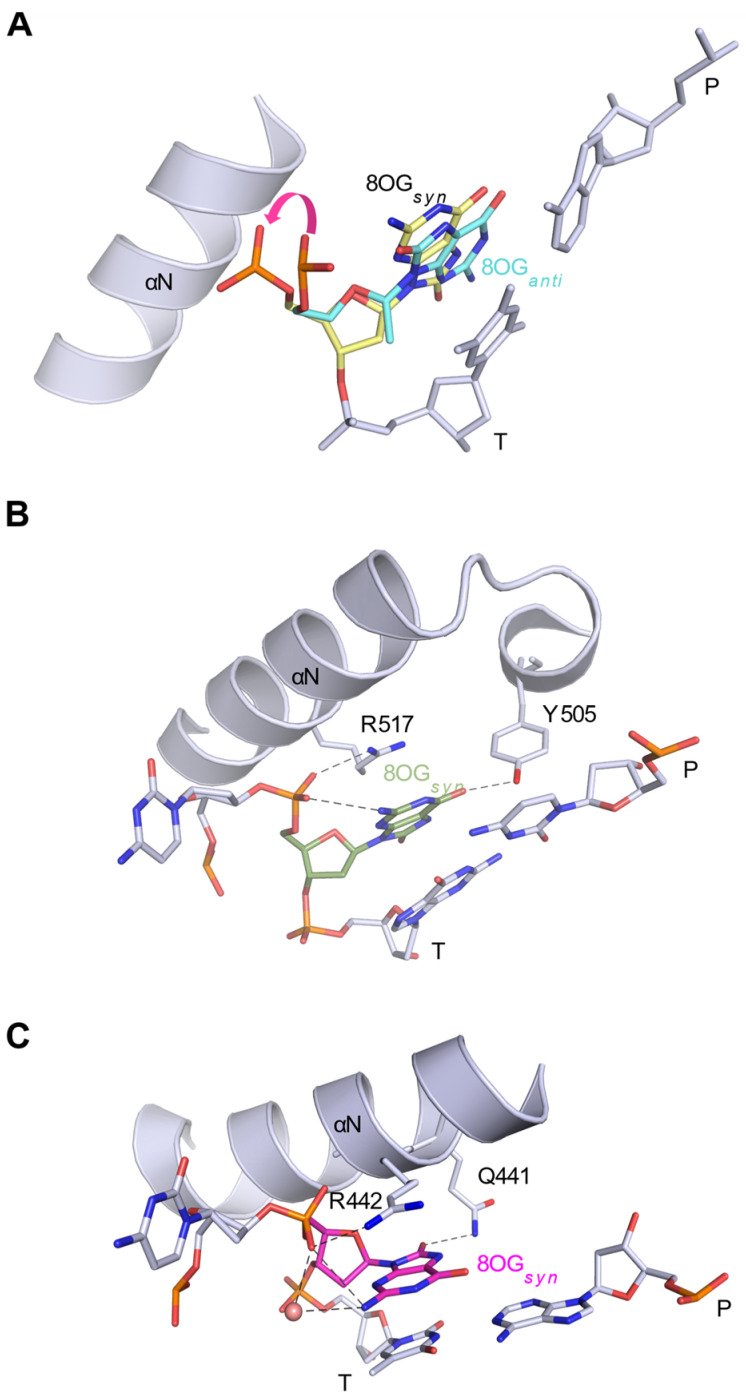
Structures of 1nt-gapped DNA substrates containing 8OG in the templating position. Stick representation of the DNA (unpaired 8OG, downstream template nucleotide and primer terminal base pair) bound to Pols β (**A**), λ (**B**) and µ (**C**) binary complexes. In A, the two conformations of 8OG, *syn* and *anti*, in the Pol β complex (PDB ID code 3RJE [54]), are shown in yellow and cyan, respectively. The magenta arrow indicates the rotation of the 5′-phosphate necessary to accommodate the O8 atom of the 8OG. A single conformation of the template 8OG is observed for Pols λ (PDB ID code 5IIO [34]) and μ (PDB ID code 6P1M [35]). α-helix N in the thumb subdomain is shown (gray ribbon). Side chains interacting with the template 8OG are drawn in stick representation, with putative hydrogen bonds indicated by dashed lines. P and T indicate the primer and template strands, respectively.

X-ray crystal structures of Pols β, λ and μ binary complexes with duplex DNA containing a single-nucleotide (1nt) gap with 8OG in the templating position inform how these polymerases engage a DNA substrate with oxidized guanine (Table 1, Table 2 and Table 3). They show that all three enzymes can accommodate the 8OG-containing substrate without gross distortions of the protein or the DNA, though each polymerase achieves this in its own specific manner. When bound to the lesion-containing DNA, in the absence of the incoming nucleotide, Polβ remains in an ‘open’ conformation, as when interacting with a substrate with an undamaged template nucleotide, with α-helix N of its thumb subdomain rotated away from the primer–template terminal base pair (PDB ID code 3RJE [54], Figure 2A).

The unpaired 8OG base is clearly mobile—relative to the neighboring template bases—and exhibits a markedly elevated B-factor. Consequently, the unpaired 8OG base in the Polβ binary complex is a mixture of the *anti* and *syn* conformations. In the *syn* conformation, the N2-amino group forms a hydrogen bond with the oxygen of its backbone phosphate. In comparison, accommodating the base in the *anti* conformation requires repositioning of the backbone phosphate, rotating its nonbridging oxygen away from the O8. Therefore, multiple conformations are observed not only for the base of the 8OG, but also for its backbone phosphate (Figure 2A). This is not the case when 8OG is positioned in the nascent base pair binding pocket opposite the incoming nucleotide or when it pairs with the primer terminal base, then its B-factor is similar to those of its template neighbors.

In contrast to Polβ, both Polλ and Polµ binary complexes bound to the 8OG-containing DNA are observed with the protein subdomains in the ‘closed’ conformation (PDB ID codes 5IIO [34] and 6P1M [35] Figure 2B,C). This is not surprising, as neither Polλ nor Polµ undergo large subdomain motions but remain ‘closed’ throughout the catalytic cycle [58,59]. The structures of Polλ and Polμ binary complexes are almost identical to their respective reference structures (PDB ID codes 1RZT [60] and 4LZG [59]) with unmodified DNA. The unpaired 8OG base in both the Polλ and Polµ binary complexes is accommodated solely in the *syn* conformation. It appears that the closed conformation of the enzyme, allowing for stabilizing interactions between the 8OG base and the residues in the thumb, restricts its mobility, deterring it from flipping between *syn* and *anti* conformations.

In the Polλ structure [34], the 8OG*_syn_* is stabilized not only by a hydrogen bond between the N2 in the base and a nonbridging oxygen on the phosphate, as observed in the Polβ complex, but also by interactions with Arg517 and Tyr505 in the thumb subdomain (Figure 2B). Arg517 stacks with the oxidized base and forms a hydrogen bond with another nonbridging oxygen on the phosphate, while Tyr505 forms a hydrogen bonding interaction with the C6-carbonyl of the oxidized base. However, in this inactive position, Tyr505 obstructs the entry of the incoming nucleotide and relocates to the ‘closed/active’ configuration to allow the nucleotide to bind.

Similarly, as observed in Polβ and Polλ complexes, the N2-amino group of 8OG in the Polµ binary complex [35] is involved not only in hydrogen-bonding interactions with the nonbridging oxygen of the 5′-phosphate, but also with a nearby water molecule (Figure 2C). The 8OG base in the Polµ complex, similar to the Pol λ complex, is sandwiched between its upstream template neighbor and a structurally equivalent arginine in the α-helix N of the thumb. In Polµ, Arg442 provides the stacking interactions with the oxidized base. Arg442 also forms a potential hydrogen bond with the nonbridging oxygen on the 5′-phosphate. A neighboring residue in α-helix N, Gln441, forms a hydrogen bonding interaction with O8 of the oxidized base. The conformation of Gln441, permitting it to form this interaction, has only been observed in the binary complex with the oxidized guanine. In all other Polµ structures of binary and ternary pre-catalytic complexes, Gln441 is rotated away from the templating base [59]. While Gln441 must reposition to the more frequently observed conformation to allow binding of the incoming nucleotide, its interaction with O8, stabilizing the *syn* conformation, may contribute to Polµ’s preference for A incorporation opposite 8OG.

## 4. Structural Mechanisms of Initial Template 8OG Bypass

**Figure 3:**
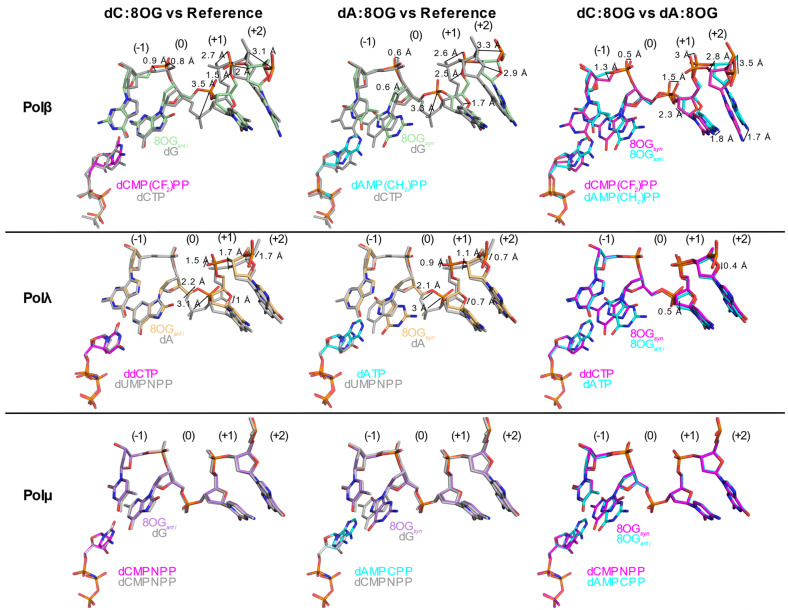
A gradient of backbone distortions observed in 1nt-gapped DNA substrates due to the presence of an oxidized guanine. Structures of pre-catalytic ternary complex structures with single-nucleotide gapped DNA containing a template 8OG are drawn in stick representation for Polβ (top row), Polλ (middle row), and Polμ (bottom row) with either cytidine (magenta, PDB ID codes 3RJI [54], 5IIJ [34], or 6P1P [35]) or adenine (cyan, PDB ID codes 3RJF [54], 5III [34], and 6P1N [35]) nucleotides. These structures (incoming dC:8OG, left column and incoming dA:8OG, middle column) were superimposed with a correctly paired reference structure (gray, PDB ID codes 4KLD [32], 2PFO [63], and 6P1V [35]). Superpositions of the incoming dC:8OG (magenta) and incoming dA:8OG (cyan) structures are shown in the right column. The naming convention is that of the incoming nucleotide:template nucleotide. The templating position is designated 0 and the downstream and upstream positions are labeled with positive and negative integers, respectively.

**Figure 4:**
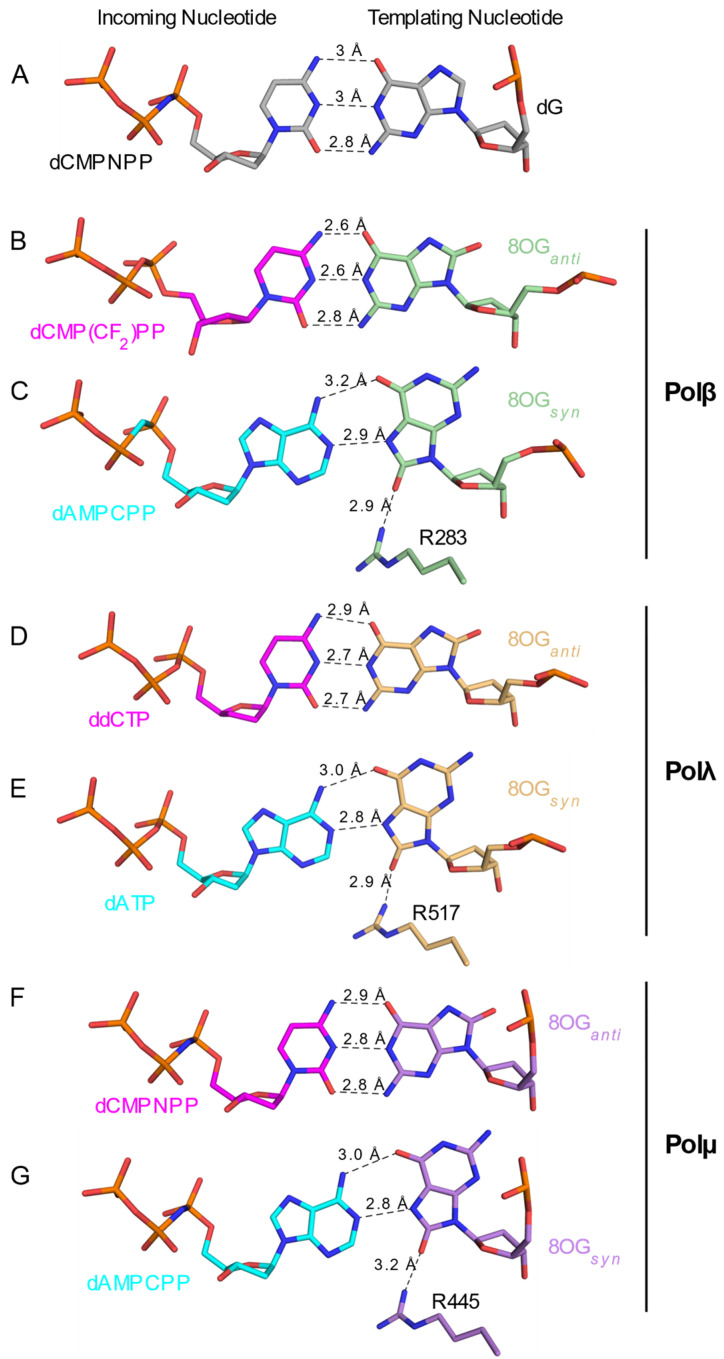
Comparison of base pairing interactions in Family X polymerases containing undamaged or oxidized template guanine nucleotides. Stick representations of the nascent base pair of either a correctly paired, undamaged guanine (**A**) bound in the active site of Polμ (PDB ID code 6P1V [35]), or oxidized template 8OG bound to Polβ ((**B**,**C**), PDB ID codes 3RJI or 3RJF [54] for correct pairs in magenta and mispairs in cyan, respectively), Polλ ((**D**,**E**), PDB ID codes 5IIJ or 5III [34] for correct pairs in magenta and mispairs in cyan, respectively), or Polμ ((**F**,**G**), PDB ID codes 6P1P or 6P1N [35] for correct pairs in magenta in mispairs in cyan, respectively). The incoming nucleotides are shown on the left and the templating guanine nucleotides on the right. Probable hydrogen bonds are drawn as black dashed lines, with distances labeled.

The Family X polymerases exhibit a gradient of movements required for catalysis^31^, which also correlates with their mechanisms for 8OG lesion bypass. These movements have been hypothesized to serve as fidelity checkpoints, impeding catalysis if specific structural and chemical requirements are not met [58,61,62]. Upon binding of an incoming nucleotide and divalent metal ions to the binary complex, Polβ undergoes movements of protein subdomains, the DNA substrate, and active site side chains in order to correctly assemble the active site and achieve a catalytically competent geometry. Polλ exhibits small-scale motions of loops, DNA and side chains. In contrast, Polμ behaves as a rigid scaffold for nucleotide incorporation. A similar gradient for structural distortions was observed in the DNA substrates of pre-catalytic ternary complex crystal structures containing a templating 8OG (Figure 3). In all ternary complex structures with a template 8OG, the incoming nucleotide always assumed an *anti* conformation, regardless of the nucleotide’s identity. With an incoming cytidine nucleotide (Figure 3, left column), the template 8OG was observed in the *anti* conformation and exhibited a canonical hydrogen-bonding pattern using the Watson–Crick edge of each base (Figure 4A,B,D,F). In the structures containing an incoming adenine nucleotide (Figure 3, middle column), the template 8OG is rotated 180° to the *syn* conformation, using its Hoogsteen edge to hydrogen bond with the adenine base (*anti*, Figure 4C,E,G). For Polβ, distortions are observed in the phosphate backbone, spanning upstream and two bases downstream of the 90° bend in the template strand (Figure 3, top row).

The most prominent disruption to backbone geometry is observed at the bend itself, shifting the phosphate between the 0 and +1 positions by 3.3–3.5 Å from its location in the correctly paired, undamaged reference structure. It has been hypothesized that either the O8 atom in the *syn* conformation, or N2 atom in the *anti* conformation, would generate a steric clash with the canonical position of the phosphate in the reference structure, likely causing this shift. Superpositions of the Polβ dC:8OG and dA:8OG structures [54] show that the backbone distortions differ. For Polλ (Figure 3, middle row), a similar backbone shift is observed between the 0 and +1 positions (3–3.1 Å). However, disruptions to the backbone in these structures involve only the region downstream of the 8OG and are decreased in magnitude, relative to those observed for Polβ. Superpositions of the Polλ dC:8OG and dA:8OG structures [34] show that the trajectories of the backbone are nearly identical, regardless of the 8OG conformation or the identity of the incoming nucleotide, and exhibit only subtle differences (Figure 3, middle row, right panel). For Polμ, however, there is no evidence of template backbone distortion with binding of either incoming cytidine or adenine nucleotides opposite the template 8OG (Figure 3, bottom row), compared to the reference structure. The 8OG is accommodated in the active site in either the *syn* or the *anti* conformations, with the N2 or O8 atoms lying within hydrogen-bonding distance (3.1–3.2 Å) of the nonbridging oxygens of the phosphate between the 0 and +1 positions. Because Polμ does not efficiently discriminate between ribo- and deoxyribonucleotides [52], crystal structures of pre-catalytic ternary complexes containing a template 8OG and correctly or incorrectly paired ribonucleotides were also solved [35], similarly showing no active site distortion [35].

**Figure 5:**
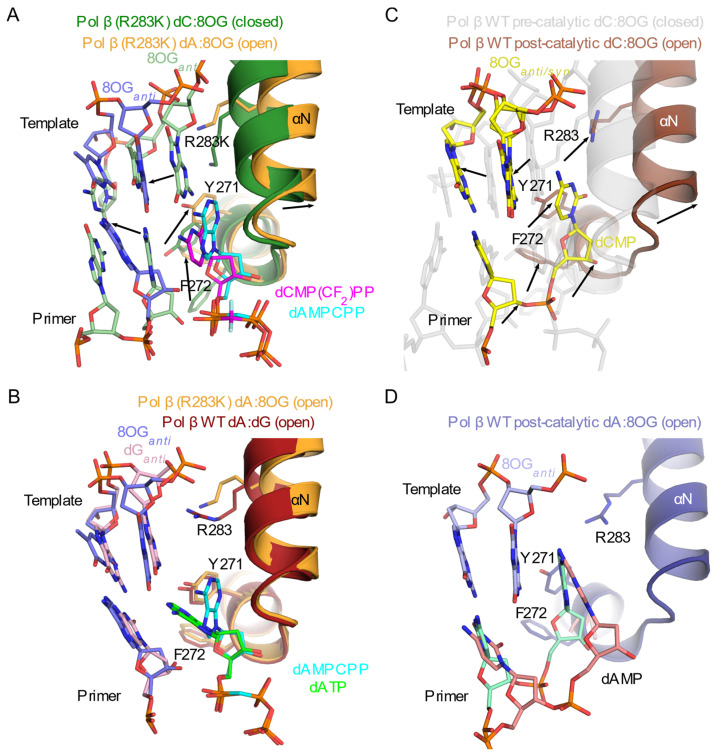
Comparison of structural rearrangements during mutagenic base pairing. (**A**). Superposition of pre-catalytic ternary complex structures of Polβ (R283K) mutant bound to single-nucleotide gapped DNA substrate with template 8OG and either incoming dCMP(CF_2_)PP (PDB ID code 4GXJ [33]; protein in dark green, DNA in light green, incoming nucleotide in magenta) or dAMPCPP (PDB ID code 4GXK [33]; protein in orange, DNA in blue, incoming nucleotide in cyan). Black arrows show structural rearrangements between closed and open conformations. (**B**). Superposition of pre-catalytic complex structures of Polβ (R283K) with dAMPCPP:8OG mispair (PDB ID code 4GXK [33]; protein in orange, DNA in blue, incoming nucleotide in cyan) and wild-type Polβ with dATP:dG mispair (PDB ID code 4LVS [32]; protein in maroon, DNA in light pink, incoming nucleotide in green). (**C**). Superposition of wild-type Polβ in pre- (PDB ID code 4RPX [55], transparent gray) and post-catalytic nicked complex of wildtype Polβ with newly incorporated dC opposite the template 8OG (PDB ID code 4RQ0 [55], protein in brown, DNA in yellow). The template 8OG is observed in both *syn* and *anti* conformations. Transitions of protein side chains and DNA nucleotides are indicated by black arrows. (**D**). Post-catalytic nicked complex of wildtype Polβ with newly incorporated dA opposite the template 8OG (PDB ID code 4RQ6 [55], protein in dark blue, DNA template strand in light blue). The template 8OG is observed in a single conformation (*anti*), while the primer strand is modeled with alternate conformations (pink and aquamarine). Secondary structural elements are shown in cartoon representation, and key active site components are drawn in stick.

Though the potential for steric clashes along the DNA backbone is one means by which polymerases attempt to differentiate between correct and mutagenic bypass of the template 8OG, these efforts can be hindered by the ‘hijacking’ of fidelity checkpoints used to detect proper hydrogen bonding interactions in the minor groove. One such example is the role played by an arginine lying in an extended conformation along the minor groove of the nascent base pair binding site (Arg283, Arg517, and Arg445 in Pols β, λ, and μ, respectively). In this position, the side chain helps select for proper base pairing by forming a hydrogen bond with any template base correctly paired opposite the primer terminus (−1 position). However, this arginine may also hydrogen bond with a template 8OG occupying the nascent base pair site in the *syn* conformation (Figure 4C,E,G). For Polβ and Polλ, this arginine only lies in the minor groove in the fully closed, catalytically assembled complex, immediately preceding chemistry. In Polμ, Arg445 is always observed in this position [59], which may contribute to mutagenic adenine insertion opposite the template 8OG, since this interaction stabilizes the 8OG*_syn_*. Interestingly, a conservative mutation of this arginine to lysine in Polβ increases dC versus dA insertion opposite the template 8OG by up to 15-fold [33]. Subsequent crystal structures of the pre-catalytic ternary complex of this Polβ (R283K) mutant with template 8OG and bound cytidine (PDB ID code 4GXJ [32]) or adenine (PDB ID code 4GXK [32]) incoming nucleotides shows that only the correctly paired dCM(CF_2_)PP-bound complex is observed in the catalytically competent closed conformation (Table 1 and Figure 5A). The binding of the incoming dAMPCPP results in an incomplete thumb subdomain closure, with a shift in the DNA template, leaving the 8OG*_anti_* base intercalated and unpaired between the primer terminal base and the incoming dAMPCPP. Active site residues remain in their open/inactive conformations. Therefore, stabilization of the 8OG*_syn_* rotation by this minor groove arginine strongly contributes to mutagenic dA insertion in the Family X polymerases. Interestingly, this configuration is similar to that observed for the wildtype protein crystallized with a 1nt gapped DNA with an undamaged templating dG and a mispaired incoming dATP, suggesting a shared mechanism for preventing mutagenic incorporation (Figure 5B).

Time-lapse crystallography studies were performed for Polβ characterizing either dCTP or dATP in crystallo incorporation opposite the template 8OG [55] (Table 1). For these studies, complete incorporation of either nucleotide opposite the 8OG were accomplished on similar time-scales—consistent with their relatively similar insertion preferences (PDB ID codes 4RQ0 and 4RQ6) [55]. However, these studies showed that, following reaction completion and pyrophosphate release, Polβ underwent a large-scale structural rearrangement to reassume the open conformation (PDB ID codes 4RQ1 and 4RQ7 [55], Figure 5C,D). The template 8OG is again unpaired in the active site, and the 3′-end of the upstream primer strand—including the newly incorporated nucleotide—becomes considerably less ordered. Opening kinetics occur on a considerably faster timescale for substrates containing the template 8OG (open after 1 h soak with MgCl_2_ [55]), compared with a substrate containing a correctly paired dCMP opposite an undamaged guanine in the nascent base pair binding site (closed after 11 h soak with MgCl_2,_ PDB ID code 4KLM [32]). In direct contrast, post-catalytic structures of Polμ (Table 3) with either cytidine or adenine nucleotides (deoxyribo- or ribonucleotides, PDB ID codes 6P1O, 6P1Q, 6P1S, 6P1U [35]) show that adenine insertion opposite the template 8OG is more efficient than that of cytidine on the same timescale, consistent with its strong preference for incorporation A rather than C. Additionally, allowing the reactions to proceed *in crystallo* for up to 46 h yielded no overt structural rearrangements. To date, post-catalytic crystal structures of template 8OG bypass by Polλ have not yet been published.

## 5. Better Late Than Never: Preventing Extension from Mutagenic 8OG Mispairs

**Figure 6:**
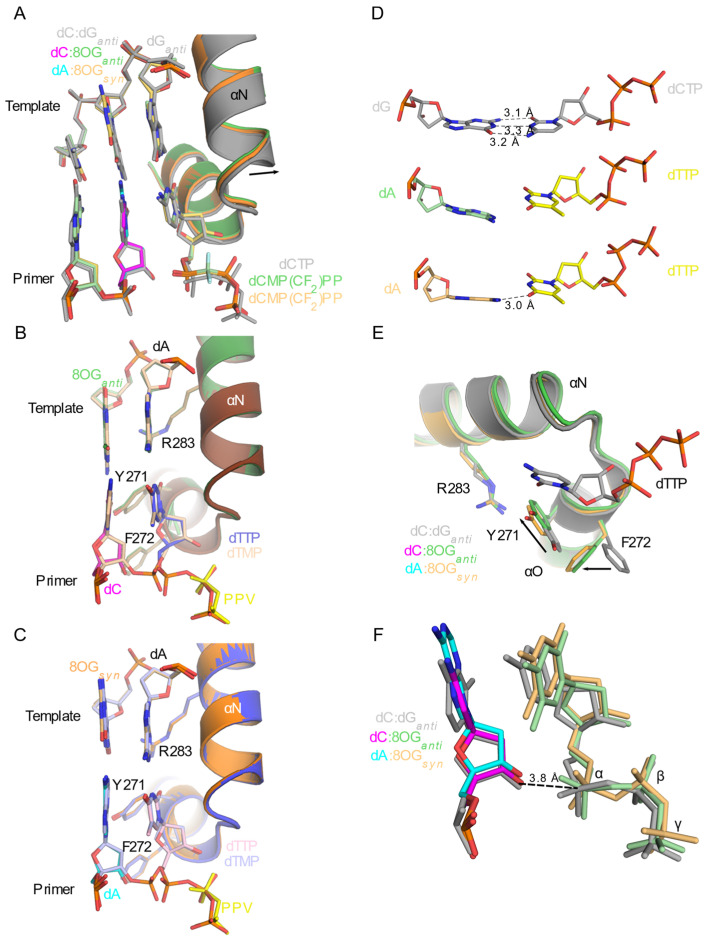
Extension from a template 8OG opposite the primer terminus by Polβ. (**A**). Superposition of Polβ pre-catalytic ternary complex structures containing an undamaged (dG, gray) or oxidized template 8OG (green or khaki) opposite the primer terminus (gray for dCMP:dG, PDB ID code 4KLD [32]; magenta for dCMP:8OG*_anti_*, PDB ID code 3RJK [54]; cyan for dAMP:8OG*_syn_*, PDB ID code 3RJH [54]) and an incoming cytidine nucleotide opposite the undamaged dG templating nucleotide in the nascent base pair binding site. Secondary structural elements are shown in cartoon representation, and key active site components are drawn in stick. (**B**). Superposition of the pre- (protein and DNA in green, incoming dTTP in slate blue, PDB ID code 5VRX [57]) and post-catalytic (protein in brown, DNA in wheat, pyrophosphate leaving group in yellow, PDB ID code 5VS0 [57]) complexes. The template 8OG lies at the −1 position, adopting the *anti* conformation opposite the primer terminal dC (magenta in pre-catalytic structure). (**C**). Superposition of the pre- (protein in orange and DNA in orange, incoming dTTP in light pick, PDB ID code 5VS2 [57]) and post-catalytic (protein in slate blue, DNA in light blue, pyrophosphate leaving group in yellow, PDB ID code 5VS4 [57]). The template 8OG lies at the −1 position, adopting the *syn* conformation opposite the primer terminal dA (cyan in pre-catalytic structure). (**D**). Comparison of the nucleotides in the nascent base pair binding pocket in the dC:dG reference structure (PDB ID code 4KLD [32], gray, top) versus those of the nonplanar dTTP:dA conformations with either primer terminal dC:8OG*_anti_* (PDB ID code 5VRW [57], middle) or dA:8OG*_syn_* (PDB ID code 5VS1 [57], bottom). Hydrogen bonds are indicated by black dashed lines. Comparison of Polβ active site residue positions (**E**) and incoming nucleotide geometry (**F**) in the undamaged reference structure (PDB ID code 4KLD [32], gray) versus those containing the nonplanar incoming dTTP, extending from the template 8OG at the −1 position (dC:8OG*_anti_* in green, PDB ID code 5VRW; dA:8OG*_syn_* in orange, PDB ID code 5VS1 [57]).

Pols β and λ are capable of processive incorporation on gapped DNA substrates [64,65], which is essential for their roles in long-patch BER. It is therefore important to understand not only how they bypass 8OG lesions in the template strand, but also how they extend from the resulting correct or incorrect pairings once they have translocated from the nascent base pair binding site to the −1 position. As described above, Polβ assumes an open conformation after incorporation opposite the template 8OG, which was originally postulated to serve as a mechanism to prevent further extension from the 8OG lesion [55]. However, subsequent X-ray crystallography studies (Table 1) demonstrated that Polβ co-crystallized in binary complex with a 1nt gapped DNA substrate containing a template 8OG lesion at the −1 position, either correctly or incorrectly paired with dCMP or dAMP, respectively. These crystals were then soaked with a correctly paired incoming nucleotide (dCMP(CF_2_)PP) opposite an undamaged template nucleotide (dG). The resulting ternary complex structures structure (PDB ID codes 3RJH and 3RJK [54]) are observed in the closed conformation, nearly indistinguishable from the correctly paired, undamaged reference (PDB ID code 4KLD [32], RMSD of 0.8–0.9 over 320–322 Cα atoms). As expected, the template 8OG adopted the *anti* conformation when correctly paired opposite the primer terminal dCMP, and the *syn* conformation when mispaired opposite the dAMP (Figure 6A).

The proposed steric clashes observed when the 8OG occupies the nascent base pair binding site are likely generated due to the 90° bend in the template backbone and are not evident after 8OG has translocated to the −1 position. *In crystallo* post-catalytic nicked complexes were not reported in this study, but were subsequently characterized via time-lapse crystallography (Table 1) [57]. This study reported two distinct conformations for the incoming nucleotide in the nascent base pair binding site. In one structure, the incoming nucleotide is observed in a catalytically relevant conformation (PDB ID codes 5VRX and 5VS2 [57]) nearly indistinguishable from those reported by Batra et al. (RMSDs of less than 0.4 Å over 273–307 Cα atoms), where the incoming base lies planar to the undamaged template nucleotide (Figure 6B,C) [54]. Time-lapse *in crystallo* incorporation reveals that the incoming dTTP is efficiently inserted opposite the templating dA, extending from either dC:8OG*_anti_* or dA:8OG*_syn_*, and occurs with no overt structural distortions during catalysis. It is unknown whether a similar reopening transition would occur, as was observed during template 8OG bypass, since the time-lapse study did not include soaking time points longer than 120 s. Extension from the primer terminal dA:8OG*_syn_* mispair occurs on a slightly slower timescale (120 s) than that of the correctly paired dC:8OG*_anti_* (80 s). These results are consistent with pre-steady state kinetic assays performed on the same substrate, which suggest that extension from a primer base paired with template 8OG is less efficient (18 to 850-fold) than initial bypass. Furthermore, extension from the correctly paired dC:8OG*_anti_* is favored 15-fold over extension from the mispaired dA:8OG*_syn_* [57].

One possible explanation for the reduction in catalytic efficiency for extension from the 8OG lesion versus that of the initial bypass is the observation of a second, apparently nonproductive conformation by Reed et al. [57]. In these structures, the incoming dTTP nucleotide exhibits an orientation where the base is rotated nonplanar to the template nucleotide. This conformation is similar in both primer terminal dC:8OG*_anti_* and the mispaired dA:8OG*_syn_* structures (PDB ID codes 5RVW and 5VS1 57, Figure 6D and Table 1). In these structures, the incoming nucleotide exhibits weaker electron density and increased thermal motion. The nonplanar orientation abolishes the canonical Watson–Crick hydrogen-bonding pattern—only a single hydrogen bond is observed in the mispaired primer terminal dA:8OG*_syn_* complex. Interestingly, a comparison of these structures to the catalytically relevant conformation demonstrates that the polymerase displays many, though not all, of the classical features of closed/active conformation (Figure 6E). The thumb subdomain has rotated toward the nascent base pair binding site and Arg283 extends into the minor groove. However, Tyr271 and Phe272 have not adopted their canonical closed/active conformations. Small distortions are observed in the triphosphate moieties of the nonplanar dTTP nucleotides, relative to that of the undamaged reference structure (PDB ID code 4KLD [32]) (Figure 6F). The primer terminal sugar adopts the C3′-*endo* sugar pucker, placing the 3′-OH in the equatorial position, which lies 3.8 Å from the α-phosphate of the incoming nucleotide in each nonplanar 8OG extension structure and in that of the undamaged reference structure. Reed et al. suggest that the nonplanar, nonproductive binding mode may exist in equilibrium with the catalytically relevant Watson–Crick conformation, which could decrease the efficiency of extension from the 8OG opposite the primer terminus [57]. However, it should be noted that the extent to which 8OG extension is impaired is a matter of some debate, since the previous study by Batra et al. observed similar catalytic efficiencies for extension and bypass [54]. Furthermore, the efficiency of the extension was nearly identical, regardless of which primer terminal pairing was present [54]. Disparities in technique (pre-steady-state versus steady-state kinetics) and sequence context could certainly influence these results, since the identity of the incoming nucleotide was different in each case.

**Figure 7:**
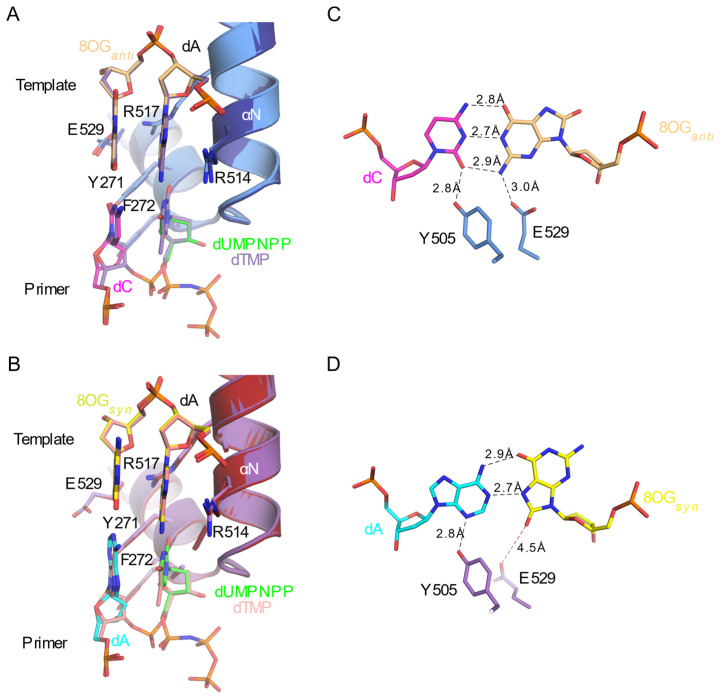
Extension from a template 8OG opposite the primer terminus by Polλ. (**A**). Superpositions of the pre- (protein in light blue, DNA in light orange, primer terminal dC in magenta, and incoming dUMPNPP in green, PDB ID code 5IIN [34]) and post-catalytic (protein in dark blue, DNA in purple, PDB ID code 5IIK [34]) complexes of dC:8OG*_anti_* bound to Polλ. (**B**). Superpositions of the pre- (protein in purple, DNA in yellow, primer terminal dA in cyan, and incoming dUMPNPP in green, PDB ID code 5IIM [34]) and post-catalytic (protein in maroon, DNA in pink, PDB ID code 5IIL [34]) complexes of dA:8OG*_syn_* bound to Polλ. Comparisons of the primer terminal base pairing and interactions with either the dC:8OG*_anti_* (**C**) or dA:8OG*_syn_* (**D**) primer termini. Nucleotides and protein side chains are colored as in (**A**) or (**B**), respectively. Hydrogen bonding interactions and their respective distances are shown as black dashed lines. The Glu529 interaction with the 8OG base that is lost in the *syn* conformation is indicated by the red dashed line.

Crystal structures and kinetic characterization are also available for Polλ’s extension from a template 8OG opposite the primer terminus [34] (Table 2). Steady-state kinetic assays suggest that, although Polλ bypasses the template 8OG in the nascent base pair binding site, incorporating dCTP or dATP with relatively equal efficiency, it shows an approximately 10-fold preference for extending from the correctly paired dC:8OG*_anti_* at the primer terminus, over that of the mispaired dA:8OG*_syn_*. Analysis of pre-catalytic ternary complexes with either dC:8OG*_anti_* or dA:8OG*_syn_* at the primer terminus and a correctly paired incoming nucleotide (dUMPNPP) opposite an undamaged template nucleotide (dA) reveal no structural distortions of the phosphodiester backbone due to the presence of the 8OG at the −1 position. The template 8OG adopts the *anti* or *syn* conformations when paired with dC or dA, respectively (Figure 7A,B).

Post-catalytic structures for these complexes are also available, though it should be noted that these DNA substrates were pre-annealed, rather than having the reaction proceed *in crystallo*. Comparison of the pre- and post-catalytic structures demonstrates that the structures are nearly indistinguishable, regardless of the identity of the pairing at the primer terminus, and that no structural distortions are apparent before or after catalysis (RMSDs of less than 0.2 Åover 273–304 Cα atoms). Detailed analysis of the dC:8OG*_anti_* or dA:8OG*_syn_* extension structures reveals a subtle, yet crucial difference in the active site of the dC:8OG*_anti_*, compared dA:8OG*_syn_* complex. When the template 8OG at the −1 position adopts the *anti* conformation, paired to the primer terminal dC, the side chain of Glu529 hydrogen bonds with the N2 atom of the 8OG base (Figure 7C). However, when the 8OG adopts the *syn* conformation, mispaired to the primer terminal dA, the Glu529 side chain swings away from the 8OG, lying too far from the base for the equivalent hydrogen bond to occur (Figure 7D). Alanine substitution of Glu529 entirely abolishes the 10-fold discrimination against extension from the mispaired primer terminal dA:8OG*_syn_*, suggesting that the hydrogen bond with the 8OG in the *anti* conformation plays a pivotal role in favoring extension from the correct pairing [34].

## 6. Conclusions

Family X polymerases participate in DNA repair, conducting short gap-filling synthesis. Polβ is the primary polymerase involved short- and long-patch BER where Polλ has been shown to play a backup role. Polλ has been implicated in MUTYH-initiated long-patch BER, and like Polμ, also participates in DSB repair by NHEJ. The structures discussed here show how the three polymerases engage gapped DNA substrates containing a template 8OG, a common oxidative lesion, that may be encountered by the polymerases during repair processes. As presented, the nascent base pair binding pockets of all three polymerases accommodate both the *anti* and *syn* conformations of 8OG. All three enzymes use an arginine residue in the thumb subdomain that normally interacts with the minor groove side of an unmodified template nucleotide to ensure correct pairing. This fidelity checkpoint is ‘hijacked’ to stabilize the 8OG in the *syn* conformation, thus enabling mutagenic bypass. These polymerases show relatively robust incorporation opposite 8OG. Both, Pols β and λ incorporate dA and dC opposite 8OG with relatively equal efficiencies that are not dissimilar from that of dC incorporation opposite an unmodified template dG. Pol µ however, incorporates only adenine opposite 8OG with an efficiency similar to that of cytidine opposite undamaged dG. An additional checkpoint can occur at the extension step to discourage polymerization from a mispaired primer terminus opposite the 8OG. As shown for Polλ, the fidelity of 8OG bypass during long-patch BER may be enhanced at this step—whether this strategy may be also utilized by Polβ remains a matter of some debate. Successful repair of DNA damage depends on the efficient, sequential action of enzymes in the repair pathway. Stalling of the repair process, for example by inefficient gap filling, could result in the accumulation of toxic repair intermediates that may be detrimental to the cell. Therefore, efficient nucleotide incorporation opposite 8OG—albeit at the potential cost of fidelity—may be beneficial for the repair process. The most noticeable example of such sacrifice of fidelity is synthesis by Pol µ during NHEJ. When using 8OG as a template, Polµ predominantly incorporates dATP and likely ATP, the most abundant nucleotide in the cell, therefore providing for efficient subsequent ligation by LigIV. The misincorporated nucleotides, whether during NHEJ or BER, can go through another round of repair to correct the error.

## Figures and Tables

**Table 1:** X-ray crystal structures of template 8OG bypass and extension by Polβ.

PDB ID	Incoming Nucleotide	Resolution (Å)	Complex	Primer:Template (*syn*/*anti*)	Notes
Template 8OG binary (0 position)					
3RJE [54]		2.1	binary	8OG (*syn/anti* mixture)	
4GXI [33]		1.95	binary	8OG (*syn/anti* mixture)	Mutant R283K
Initial bypass of template 8OG at 0 position (pre-catalytic to post-catalytic)					
4RQ3 [55]	dATP	2.0	pre-catalytic	dA*_anti_*:8OG*_syn_* paired	
3RJF [54]	dAMPCPP	2.3	pre-catalytic	dA*_anti_*:8OG*_syn_* paired	
4RPX [55]	dCTP	1.9	pre-catalytic	dC*_anti_*:8OG*_anti_* paired	
1MQ3 [27]	dCTP	2.8	precatalytic	dC*_anti_*:8OG*_anti_* paired	
4GXJ [33]	dCMP(CF_2_)PP	2.2	pre-catalytic	dC*_anti_*:8OG*_anti_* paired	Mutant R283K
4F5O [56]	dCMP(CF_2_)PP	2.0	pre-catalytic	dC*_anti_*:8OG*_anti_* paired	
3RJI [54]	dCMP(CF_2_)PP	2.3	pre-catalytic	dC*_anti_*:8OG*_anti_* paired	
4GXK [33]	dAMPCPP	2.0	pre-catalytic	dA*_anti_*:8OG*_syn_* paired	Mutant R283K
4RQ4 [55]	dATP	2.1	incomplete catalysis 30 s	dA*_anti_*:8OG*_syn_* paired	
4RQ5 [55]	dATP	2.3	incomplete catalysis 60 s	dA*_anti_*:8OG*_syn_* paired	
4RQ6 [55]	dATP	2.3	post-catalytic 80 s	dA*_anti_*:8OG*_syn_* paired	
4RQ7 [55]	dATP	2	post-catalytic 1 h	dA (*syn/anti* mixture):8OG*_anti_* unpaired	
4RQ8 [55]	dATP	2	post-catalytic 35 s	dA*_anti_*:8OG*_syn_* paired	
1MQ2 [27]	dAMP	3.1	Pre-catalytic mimic	dA*_anti_*:8OG*_anti_* unpaired	
4RPY [55]	dCTP	1.9	incomplete catalysis 30 s	dC*_anti_*:8OG*_anti_* paired	
4RPZ [55]	dCTP	2.2	incomplete catalysis 60 s	dC*_anti_*:8OG*_anti_* paired	
4RQ0 [55]	dCTP	2.2	post-catalytic 80 s	dC*_anti_*:8OG_anti_ paired	
4RQ1 [55]	dCTP	2.7	post-catalytic	dC*_anti_*:8OG (*syn/anti* mixture) unpaired	
4RQ2 [55]	dCTP	2.2	post-catalytic	dC*_anti_*:8OG*_anti_* paired	
Extension bypass binary (−1 position)					
3RJG [54]		2.0	binary	dA*_anti_*:8OG*_syn_*	
3RJJ [54]		2.0	binary	dC*_anti_*:8OG*_anti_* paired	
Extension from template 8OG at −1 position (pre-catalytic to post-catalytic)					
5VS1 [57]	dTTP	2.5	pre-catalytic	dA*_anti_*:8OG*_syn_* paired	nascent base pair non-planar
5VS2 [57]	dTTP	2.3	pre-catalytic	dA*_anti_*:8OG*_syn_* paired	
3RJH [54]	dCMP(CF_2_)PP	2.2	pre-catalytic	dA*_anti_*:8OG*_syn_* paired	
5VRW [57]	dTTP	2.6	pre-catalytic	dC*_anti_*:8OG*_anti_* paired	nascent base pair non-planar
5VRX [57]	dTTP	2.2	pre-catalytic	dC*_anti_*:8OG*_anti_* paired	
3RJK [54]	dCMP(CF_2_)PP	2.1	pre-catalytic	dC*_anti_*:8OG*_anti_* paired	
5VS3 [57]	dTTP	1.7	incomplete catalysis 90 s	dA*_anti_*:8OG*_syn_* paired	
5VS4 [57]	dTTP	1.9	post-catalytic 120 s	dA*_anti_*:8OG*_syn_* paired	
5VRY [57]	dTTP	1.9	incomplete catalysis 20 s	dC*_anti_*:8OG*_anti_* paired	
5VRZ [57]	dTTP	2.1	incomplete catalysis 40 s	dC*_anti_*:8OG*_anti_* paired	
5VS0 [57]	dTTP	2.1	post-catalytic 80 s	dC*_anti_*:8OG*_anti_* paired	

**Table 2:** X-ray crystal structures of template 8OG bypass and extension by Polλ.

PDB ID	Incoming Nucleotide	Resolution (Å)	Complex	Primer/Template (*syn/anti*)
Template 8OG binary (0 position)				
5IIO [34]		2.1	binary	8OG*_anti_*
Initial bypass of template 8OG at 0 position (pre-catalytic to post-catalytic)				
5III [34]	dATP	1.8	pre-catalytic	dA*_anti_*:8OG*_syn_* paired
5IIJ [34]	dCTP	1.7	pre-catalytic	dC*_anti_*:8OG*_anti_* paired
Extension from template 8OG at −1 position (pre-catalytic and post-catalytic)				
5IIM [34]	dUMPNPP	1.9	pre-catalytic	dA*_anti_*:8OG*_syn_* paired
5IIN [34]	dUMPNPP	2.1	pre-catalytic	dC*_anti_*:8OG*_anti_* paired
5IIL [34]		2.0	post-catalytic	dA*_anti_*:8OG*_syn_* paired
5IIK [34]		2.0	post-catalytic	dC*_anti_*:8OG*_anti_* paired

**Table 3:** X-ray crystal structures of template 8OG bypass by Polμ.

PDB ID	Incoming Nucleotide	Resolution (Å)	Complex	Primer/Template (*syn/anti*)
Template 8OG binary (0 position)				
6P1M [35]		1.65	binary	8OG*_syn_*
Initial bypass of template 8OG at 0 position (pre-catalytic to post-catalytic)				
6P1N [35]	dAMPNPP	1.6	pre-catalytic	dA*_anti_*:8OG*_syn_* paired
6P1P [35]	dCMPNPP	1.75	pre-catalytic	dC*_anti_*:8OG*_anti_* paired
6P1O [35]	dATP	1.65	post-catalytic	dA*_anti_*:8OG*_syn_* paired
6P1Q [35]	dCTP	1.9	post-catalytic	dC*_anti_*:8OG*_anti_* paired
6P1R [35]	rAMPNPP	1.7	pre-catalytic	rA*_anti_*:8OG*_syn_* paired
6P1T [35]	rCMPCPP	1.7	pre-catalytic	rC*_anti_*:8OG*_anti_* paired
6P1S [35]	rATP	1.75	post-catalytic	rA*_anti_*:8OG*_syn_* paired
6P1U [35]	rCTP	1.75	post-catalytic	rC*_anti_*:8OG*_anti_* paired
